# CsbZIP2-miR9748-CsNPF4.4 Module Mediates High Temperature Tolerance of Cucumber Through Jasmonic Acid Pathway

**DOI:** 10.3389/fpls.2022.883876

**Published:** 2022-04-28

**Authors:** Lan Li, Guangling Chen, Mingzhu Yuan, Shirong Guo, Yu Wang, Jin Sun

**Affiliations:** College of Horticulture, Nanjing Agricultural University, Nanjing, China

**Keywords:** cucumber, high temperature stress, miR9748, jasmonic acid, CsbZIP2, CsNPF4.4

## Abstract

High temperature stress seriously affects the growth of cucumber seedlings, and even leads to a decline in yield and quality. miRNAs have been shown to be involved in regulating the response to stress in plants, but little is known about its effects on cucumber high temperature stress tolerance. Here, we found that high temperature stress induced the expression of miR9748 in cucumber. Overexpression of cucumber miR9748 in Arabidopsis improved high temperature tolerance. Transcriptome analysis revealed that miR9748 might mediate high temperature tolerance through plant hormone signal pathway. 5′ RNA ligase-mediated rapid amplification of cDNA ends (5′ RLM-RACE) and transient transformation technology demonstrated that *CsNPF4.4* was the target gene of miR9748. *CsNPF4.4* overexpression plants decreased high temperature tolerance accompanied by reducing the content of jasmonic acid (JA), but alleviated by foliar application of methyl jasmonate, indicating that *CsNPF4.4* negatively regulated high temperature stress tolerance through inhibition JA signal pathway. Furthermore, high temperature stress also increased the expression level of *CsbZIP2*. Yeast one-hybrid and dual-luciferase assays showed that CsbZIP2 directly bound to the promoter of *MIR9748* to induce its expression. Taken together, our results indicated that CsbZIP2 directly regulated miR9748 expression to cleave *CsNPF4.4* to mediate high temperature tolerance through JA pathway.

## Introduction

The protected cultivation area of cucumber (*Cucumis sativus* L.) is the second in China, which reached 105 million hectares in 2018 (Ji et al., 2020). With the global warming, high temperature has become a key limited effect for protected cucumber cultivation in China. Studies have shown that high temperature inhibits the growth and development of cucumber plants, as indicated by inhibited the bioaccumulation, decreased chlorophyll content, increased the lipid peroxidation level, delayed flowering, and appeared dropping flower and fruit (Zhou et al., 2016; Wang et al., 2018a; Chen et al., 2021b). Cucumber plants have evolved a series of physiological and molecular mechanisms in response to high temperature stress, such as increasing the content of proline, activating antioxidant defense system, inducing the expression of heat shock proteins, and regulating plant hormone signal pathways (Wei et al., 2019; He et al., 2020; Chen et al., 2021b). Furthermore, calcium signal, transcription factors, and post-transcription regulation also mediate cucumber high temperature stress tolerance (Zhou et al., 2013; Xu et al., 2015; Yu et al., 2018; Wang et al., 2018b, 2020a).

Plant miRNA is a kind of non-coding RNA and regulates the expression of target mRNA by cleavage or translation inhibition (Chen et al., 2018). miRNA is transcribed by RNA Polymerase II into primary miRNAs (pri-miRNA) with typical stem-loop structure. Subsequently, pri-miRNA is cleaved twice by DICER-LIKE1 (DCL1) to produce miRNA/miRNA^*^ double strands. The methylated double strands are transported from the nucleus to the cytoplasm by HASTY. In the cytoplasm, miRNA is loaded into ARGONAUTE 1 protein to form an active RNA-induced silencing complex, and miRNA^*^ is degraded (Wang et al., 2020b). miRNA plays critical roles in plant adaptation to high temperature stress (Wang et al., 2018b; Bhogireddy et al., 2021). miR156 is induced by high temperature stress to regulate the expression of *SPL* gene and promote the continuous expression of genes in response to high temperature stress (Cui et al., 2014; Stief et al., 2014). In contrast to miR156, the expression of miR172 is downregulated under high temperature stress, while the expression of target genes *TARGET OF EAT1* (*TOE1*) and *TOE2* is upregulated (May et al., 2013; Li et al., 2014). High temperature rapidly increases the expression level of miR398, which positively regulates heat stress tolerance through downregulation the expression of *CSD1* (copper/zinc superoxide dismutase), *CSD2,* and *CCS* (a copper chaperone for CSD1 and CSD2; Guan et al., 2013). In addition, miRNA might mediate spermidine-induced high temperature stress tolerance in cucumber (Wang et al., 2018b). These results demonstrate that miRNA can help plants adapt to high temperature stress by regulating the expression of target genes.

miR9748 is a particularly conservative miRNA family, which mediates plant growth and stress response. It has been shown that *EIN3* is targeted by miR9748 in radish to regulate anthocyanin accumulation by mediating sucrose signal pathway (Sun et al., 2017). miR9748 also regulates the formation of adventitious roots in lotus by affecting the expression of downstream genes and participating in the metabolic process of brassinosteroid and upregulating the expression of *BRI1* (Cheng et al., 2019). In addition, miR9748 participates in regulation the expression of *MYC2* and *HSP90* in *Astragalus chrysochlorus* (Cakir et al., 2016), indicating that it might mediates plants response to stress. In our previous work, we constructed the competing endogenous RNA (ceRNA) networks of long non-coding RNAs (lncRNAs), circular RNAs (circRNAs), miRNAs, and mRNAs under high temperature stress, and found that miR9748 is the central molecule of cucumber heat stress response ceRNA network (He et al., 2020), but the response mechanism of miR9748 to high temperature stress has not been understood. Here, we found that transcription factor CsbZIP2 bound to the promoter of *MIR9748* to induce its expression to further degrade *CsNPF4.4*. Overexpression of miR9748 in Arabidopsis enhanced high temperature stress tolerance, but *CsNPF4.4* overexpression plants were hypersensitivity to high temperature stress, along with inhibiting the genes expression related to jasmonic acid (JA) synthesis and decreasing the JA content. However, foliar application of methyl jasmonate (MeJA) to *CsNPF4.4* overexpression plants increased high temperature stress tolerance. Thus, CsbZIP2 directly regulated miR9748 expression to cleave *CsNPF4.4* to mediate high temperature stress tolerance through JA pathway.

## Materials and Methods

### Plant Materials and Treatments

Cucumber (*Cucumis sativus* L, Jinchun No. 2) was used in this experiments, and the seeds were purchased from Tianjin Kernel Cucumber Research Institute (Tianjin, China). The germinated seeds were sown in plastic pots (10 cm × 7 cm × 8 cm) filled with peat and vermiculite (2,1, v:v). The growth conditions were maintained as follow: 25/18°C day/night, 60–70% relative air humidity, 300 μmol m^−2^ s^−1^ photosynthetic photon flux density (PPFD), and 14/10 h light/dark cycle. When the third leaves were fully expanded, the seedlings were treated with 42/32°C (day/night) as high temperature stress. The leaf samples were harvested at 0, 1, 2, 4, 6, 12, 24, and 48 h and frozen in liquid nitrogen and stored in −80°C.

### Construction of *MIR9748* and *CsNPF4.4* Overexpression Plants

A 430 bp sequence containing the precursor of miR9748 was synthesized by General Biological Systems Co., LTD. (Chuzhou, China), and inserted into pFGC1008 vector. The full-length coding DNA sequence (CDS) of *CsNPF4.4* was amplified using cucumber cDNA as template with the specific primers (Supplementary Table 1). The PCR fragment was ligated into the plant transformation vector pFGC1008 using the ClonExpress II One Step Cloning Kit (Vazyme, Nanjing, China). The constructed pFGC1008-*MIR9748* and pFGC1008-*CsNPF4.4* plasmids were transformed into *Agrobacterium tumefaciens* strain EHA105 and transformed Arabidopsis Col-0 wild-type (WT) plants using floral dip method (Clough and Bent, 1998). The transformed plants were selected and verified using qPCR and the homozygous lines of the T_3_ progeny were used for high temperature stress as the same method of cucumber.

### Transcriptome Analysis of *MIR9748* Transgenic Plants

Arabidopsis leaves were collected at 8 h of high temperature stress and the total RNA was extracted from the leaves of WT and miR9748 overexpression (OE3) Arabidopsis plants using TRIzol reagent (Invitrogen, Carlsbad, CA, United States). The RNA quality was measured by a Nanodrop 2000 (Thermo Fisher Scientific, Rockford, IL, United States), and the high quality and integrity RNA samples were selected to construct RNA libraries. The quality and quantity of the library were verified using an Agilent 2,100 Bioanalyzer (Agilent Technologies, Santa Clara, CA, United States) and ABI StepOnePlus real-time PCR System (Applied Biosystems, Foster, CA, United States), respectively. Then, the libraries were sequenced on a HiSeq 2000 platform (Illumina, San Diego, CA, United States) by the BGI, Shenzhen, China. After the original readings, adapter sequences and low-quality readings were removed, all of the clean reads were mapped to the Arabidopsis reference genome using HISAT2 (V2.0.4; Kim et al., 2015). The gene expression level was calculated using the fragments per kilobase of exon per million fragments (Trapnell et al., 2012). The differentially expressed genes (DEGs) were recognized according to the false discovery rate (FDR) value less than 0.01 and |log_2_(fold change)| ≥ 2. Gene ontology (GO) analysis was performed by WEGO (Ashburner et al., 2000) and Kyoto Encyclopedia of Genes and Genomes (KEGG) pathway analysis was performed using KOBAS 2.0 (Xie et al., 2011).

### miR9748 Target Gene Prediction, and GO Pathway Enrichment Analysis

Target Finder and psRobot software were used to predict the target gene of miR9748 as previously described (Allen et al., 2005; Wu et al., 2012), and the co-predicted genes were selected as its target genes. The predicted target genes were employed for GO pathway analysis as above described.

### 5′ RNA Ligase-Mediated Rapid Amplification of cDNA Ends

To verify the cleavage relationship of miR9748 to CsaV3_5G039430, 5′ RLM-RACE was performed using the FirstChoice^™^ RLM-RACE Kit (AM1700, Invitrogen, Carlsbad, CA, United States) according to the manufacturer’s instructions. The correct PCR reaction products were cloned into pMD-19 T vector, and all positive clones were confirmed by PCR. The clones were sequenced by General Biological Systems Co., LTD. (Chuzhou, China).

### GUS Histochemical Staining Analysis

The full-length CDS of CsaV3_5G039430 was amplified with the specific primers (Supplementary Table 1) and inserted into pBI121 using the ClonExpress II One Step Cloning Kit (Vazyme, Nanjing, China) to obtain *35S*:: CsaV3_5G039430-GUS vector. For mutation 6 bases of miR9748 binding sites in CsaV3_5G039430, the CDS was amplified and inserted into pBI121 vector using the ClonExpress MultiS One Step Cloning Kit (Vazyme, Nanjing, China) to obtain *35S*:: CsaV3_5G039430M-GUS vector. The recombinant plasmids were transformed into *A. tumefaciens* strain EHA105, and transiently transformed into the leaves of *Nicotiana benthamiana* as previously described method (Wang et al., 2020c). After 2 d transformation, the leaves were stained with GUS staining kit (Solarbio, Beijing, China) and photographed.

### Subcellular Localization of CsNPF4.4

Subcellular localization of CsNPF4.4 was performed as previously described (Liu et al., 2021). The full-length CDS of *CsNPF4.4* was amplified with the primers (Supplementary Table 1) and inserted into pFGC5941-GFP vector to generate a *CsNPF4.4*-GFP fusion expression vector. Subsequently, the pFGC5941-*CsNPF4.4*-GFP and pFGC5941-GFP empty vector were transformed into *A. tumefaciens* strain EHA105, and infiltrated into the leaves of *N. benthamiana* that expresses a H2B-RFP as a marker for nucleus (Mei et al., 2020). After inoculation for 48 h, the GFP and RFP fluorescence signals were observed under an LSM800 confocal microscope (Zeiss, Oberkochen, Germany).

### Plant Hormone Treatment

For plant hormone treatment, 100 μmol MeJA was sprayed on 35 d Arabidopsis seedlings and distilled water was used as the control. After 12 h of pretreatment, high temperature treatment was performed as above described. After 2 d of treatment, the leaves of Arabidopsis were collected for determination of physiological indexes.

### Yeast One-Hybrid Assay

The yeast one-hybrid assays were performed as the method previously described (Wang et al., 2019). The promoter sequence of *MIR9748* was cloned using the specific primers (Supplementary Table 1) and inserted into the pAbAi vector. The recombinant plasmid was linearized by *Bst*B I (Thermo Fisher Scientific, Rockford, IL, United States) and transformed into Y1HGold yeast strain. The full-length CDS of *CsbZIP2*, *CsMYB44*, *CsMYCI*, *CsHBP-1b*, *CsTGA2*, *CsTGA2.2*, and *CsTGA10* was amplified with the specific primers (Supplementary Table 1) and ligated into the pGADT7 vector, respectively. The pGADT7 empty vector or pGADT7 harboring the transcription factors was transformed into the positive strains containing the bait vector, respectively, and cultured on SD/Leu solid medium containing 200 ng ml^−1^ aureobasidin A (AbA) for 3–5 d at 30°C to detect DNA–protein interactions.

### Dual-Luciferase Assay

The dual-luciferase assay was performed as previously described (Yang et al., 2021). The promoter sequence and CDS of *MIR9748* and *CsbZIP2* was amplified with specific primers (Supplementary Table 1), and inserted into the pGreenII 0800-LUC and pFGC5941-GFP vector, respectively. *A. tumefaciens* strain GV3101 (pSoup-p19) containing the indicated recombinant plasmids injected into the leaves of *N. benthamiana*. After injection for 48 h, luciferase luminescence was detected using a Tanon 5200Multi Image System (Tanon, Shanghai, China).

### RNA Extraction and Gene Expression Analysis

miRNAs were extracted from leaves of all treated samples using the miRcute miRNA Extraction Kit (Tiangen, Beijing, China). Reverse transcription of miRNA into cDNA was performed using a Mir-X miRNA first-strand Synthesis Kit (Takara, Dalian, China). The obtained cDNA was used for qPCR analysis with the TB Green Advantage qPCR Premix (Takara, Dalian, China). The *U6* gene was selected as an internal reference for standardized data, and the primers are listed in Supplementary Table 2.

Total RNA was extracted from leaves of treated samples using RNA Simple Total RNA Kit (Tiangen, Beijing, China). Total RNA was reverse transcribed into cDNA using the HiScript II Q RT SuperMix for qPCR (+gDNA wiper) Kit (Vazyme, Nanjing, China). qPCR was performed with the ChamQ SYBR qPCR Master Mix (Vazyme, Nanjing, China) on the StepOnePlus^™^ Real-Time PCR System (Applied Biosystems, United States) and the specified primers (Supplementary Table 2) were designed according to gene CDS sequence. *Actin* gene was selected as an internal control and the relative gene expression was calculated as previously described (Livak and Schmittgen, 2001).

### Chlorophyll, Proline, H_2_O_2_, Malondialdehyde Content, and Electrolyte Leakage Measurement

After 2 d of high temperature treatment, the contents of chlorophyll, proline, and H_2_O_2_ and the value of electrolyte leakage were determined. The content of chlorophyll was determined by 80% acetone extracts method (Arnon, 1949). The content of proline was determined by the method previously described (Bates et al., 1973). H_2_O_2_ content and the value of electrolyte leakage were measured as previously described (Zhang et al., 2021). The content of malondialdehyde (MDA) in leaves was determined by thiobarbituric acid method (Hodges et al., 1999).

### Determination of Abscisic Acid, JA, and Ethylene Content

0.2 g leaves of Arabidopsis seedlings were weighed and determined using ELISA kit (Shanghai Renjie Biotechnology Co., LTD.) according to the manufacturer’s instructions.

### Statistical Analysis

At least 3 independent replicates were used for each determination. Analysis of variance (ANOVA) was used to test for significance. Different letters above the bars indicate significant differences with Tukey’s test at *p* < 0.05.

## Results

### High Temperature Stress Induces the Expression of miR9748 in Cucumber Leaves

**Figure 1:**
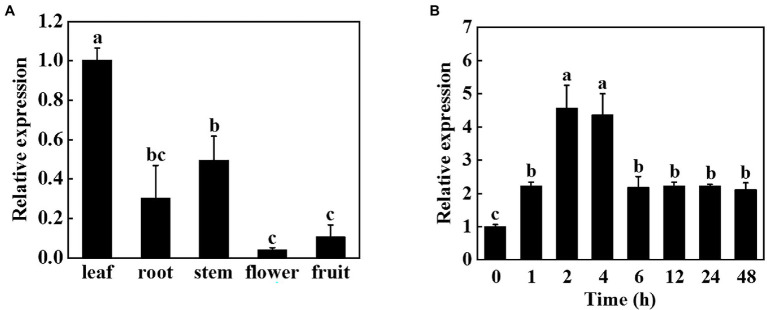
Expression patterns of cucumber miR9748 in different tissues and in response to high temperature stress. **(A)** qPCR analysis the expression of miR9748 in cucumber leaves, roots, stems, flowers, and fruits. The expression level of miR9748 in leaves was set to 1.0. **(B)** qPCR analysis the expression level of miR9748 under high temperature stress. The leaf samples were harvested at the indicated time points and analyzed by qPCR. The results represent the mean ± SD (*n* = 3). Means with the same letter did not significantly differ at *p* < 0.05 according to Tukey’s test.

To investigate the role of miR9748 in cucumber, we first analyzed its expression patterns in different tissues. Tissue expression analysis revealed that miR9748 was expressed in different tissues, with the lowest expression in flowers, the higher expression in fruits, roots, and stems, and the highest expression in leaves, suggesting that miR9748 was mainly expressed in cucumber leaves (Figure 1A). Therefore, we further analyzed the response of miR9748 in cucumber leaves under high temperature stress. The expression level of miR9748 was upregulated after high temperature treatment and reached the peak at 2 h, which was approximately 4.5 times that of 0 h (Figure 1B), indicating that the expression level of miR9748 in cucumber leaves was induced by high temperature.

### Ectopic Overexpression of miR9748 in Arabidopsis Improves High Temperature Stress Tolerance

**Figure 2:**
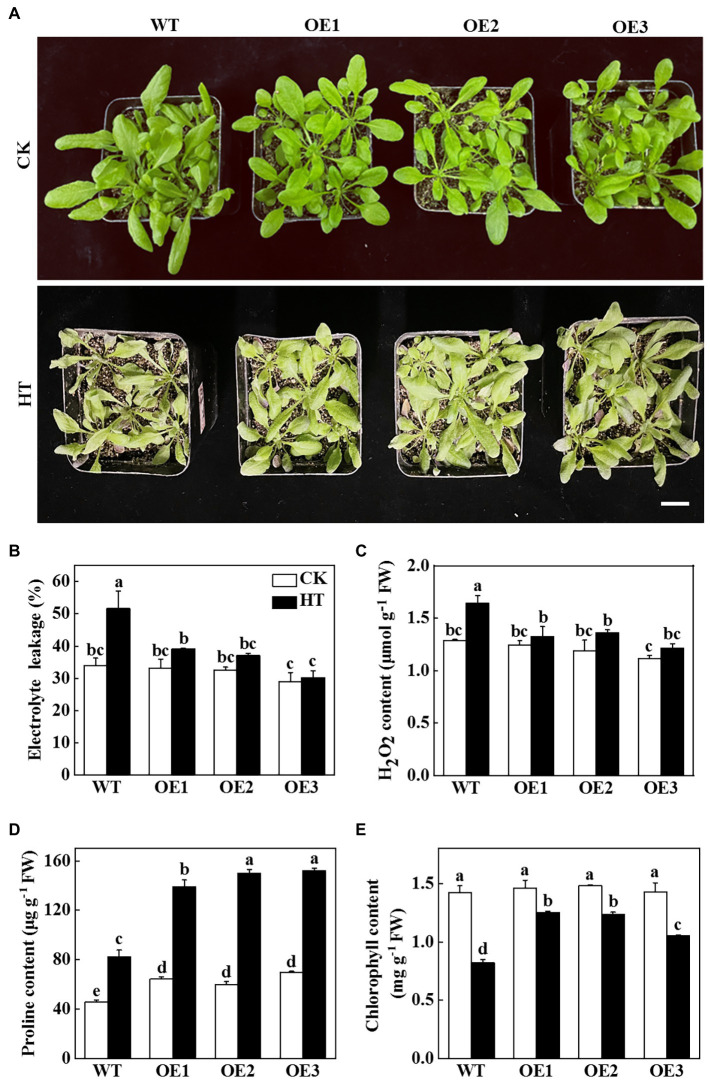
Functional analysis of miR9748 in response to high temperature stress. **(A)** Overexpression of cucumber miR9748 in Arabidopsis improved high temperature stress tolerance. Bar: 1 cm. **(B)** Electrolyte leakage. **(C)** H_2_O_2_ content in leaves. **(D)** Proline content in leaves. **(E)** Chlorophyll content. 35-d-old Arabidopsis seedlings were subjected to high temperature stress for 2 d, and the phenotype, electrolyte leakage, H_2_O_2_, proline, and chlorophyll content were measured. The results represent the mean ± SD of 3 replicates. Means with the same letter did not significantly differ at *p* < 0.05 according to Tukey’s test. CK, control; HT, high temperature; FW, fresh weight.

In order to verify whether miR9748 plays a vital role in cucumber response to high temperature stress, we predicted the precursor sequence of miR9748, and then constructed the miR9748 overexpression plants in *Arabidopsis thaliana* (OE1, OE2, and OE3). The expression level of miR9748 in overexpression plants was 4.8- to10.9-fold of WT plants (Supplementary Figure 1A). The miR9748 overexpression plant was smaller than WT plant under normal growth condition, as indicated by lower fresh and dry weight (Supplementary Figures 1B,C). After 2 d of high temperature treatment, the wilting degree of leaves in WT plants was more obvious than that of miR9748 overexpression plants (Figure 2A). High temperature treatment resulted in 38.8, 22.1, 22.3, and 20.0% decrease in the fresh weight of WT, OE1, OE2, and OE3 plants, respectively (Supplementary Figure 1B), and the dry matter accumulation decreased by 36.8, 24.4, 18.7, and 19.7%, respectively, compared with their own control plants (Supplementary Figure 1C).

To further demonstrate the role of miR9748 under high temperature stress, physiological indices of high temperature tolerance in WT and miR9748 overexpression plants were analyzed. There was no significant difference in the values of electrolyte leakage in all of the plant under optimal growth temperature, but high temperature stress induced the increase of the level of electrolyte leakage, especially in WT plants, which was 24.1 to 41.4% higher than that in miR9748 overexpression plants (Figure 2B). The content of H_2_O_2_ in WT plants was 19.5 to 26.1% higher than that in miR9748 overexpression plants under high temperature stress (Figure 2C). However, the proline and chlorophyll content of WT plants were significantly lower than that of miR9748 overexpression plants under high temperature stress (Figures 2D,E). These results suggested that overexpression of miR9748 could improve the high temperature tolerance.

### miR9748 Regulates the Expression of Genes Related to ABA, ETH, and JA Signaling Pathways

**Figure 3:**
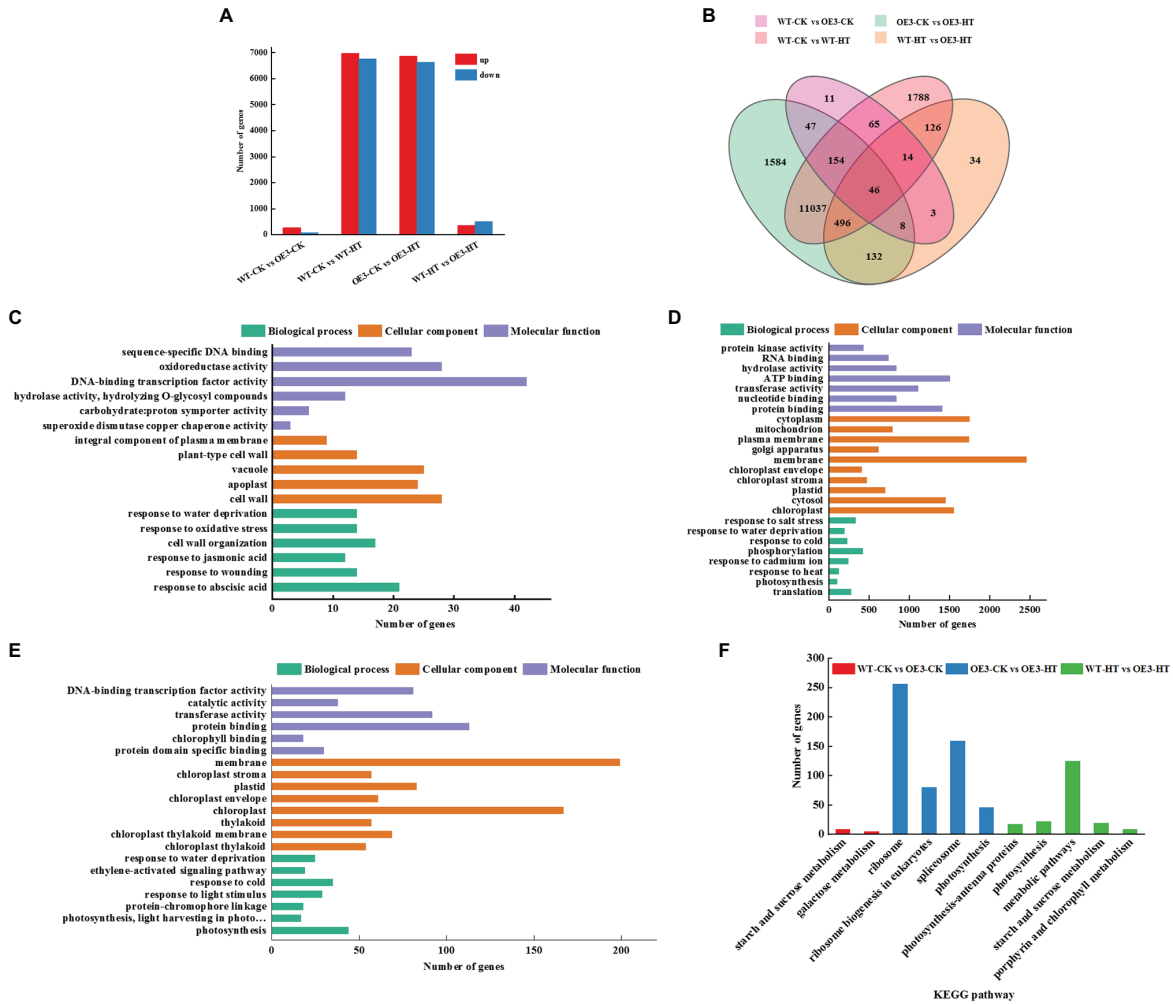
GO and KEGG pathway analysis of differentially expressed genes (DEGs) between wild-type (WT) and miR9748 overexpression (OE3) plants under high temperature stress. **(A)** The total number of upregulated and downregulated DEGs after high temperature stress. **(B)** Venn diagrams analysis of DEGs between WT and OE3 plants under high temperature stress. **(C)** GO enrichment analysis of DEGs of WT-CK vs. OE3-CK. **(D)** GO enrichment analysis of DEGs of OE3-CK vs. OE3-HT. **(E)** GO enrichment analysis of DEGs of WT-HT vs. OE3-HT. **(F)** KEGG pathway enrichment analysis of WT-CK vs. OE3-CK, OE3-CK vs. OE3-HT, and WT-HT vs. OE3-HT. CK, control; HT, high temperature.

To further explore the molecular regulatory pathways of miR9748 under high temperature stress, RNA sequencing (RNA-seq) analysis was performed on WT and OE3 plants. Illumina sequencing was performed on 12 leaf cDNA libraries (WT and OE3 plants were treated at optimal temperature and high temperature, 3 replicates per treatment). After removing the low quality, adapter contamination, and unknown high N reads from the results, 23,131 clean reads were obtained, with an average net read of 90.81% for WT-CK, 90.98% for WT-HT, and 90.87% for OE3-CK. The average net read of OE-HT library was 92.38% (Supplementary Table 3). After the clean reads were obtained, HISAT2 was used to alignment clean reads to Arabidopsis reference genome database, and the results showed that over 92.0% of the reads were uniquely mapped to the genome (Supplementary Table 4). There were 348 DEGs of WT-CK vs. OE3-CK, of which 268 DEGs were upregulated and 80 DEGs were downregulated (Figures 3A,B). There were 13,726 DEGs of WT-CK vs. WT-HT, including 6,974 upregulated and 6,752 downregulated genes (Figures 3A,B). There were 13,504 genes with significant differential expression of OE3-CK vs. OE3-HT. Among these, 6,873 genes were upregulated and 6,631 genes were downregulated (Figures 3A,B). A total of 859 DEGs of WT-HT vs. OE3-HT were obtained, of which 354 DEGs were upregulated and 505 DEGs were downregulated (Figures 3A,B). GO enrichment pathways analysis showed that these DEGs were mainly related to abscisic acid (ABA), ethylene (ETH), and JA signal pathways (Figures 3C–E), indicating that these plant hormones signaling might mediate high temperature tolerance. KEGG pathway analysis showed that DEGs were mainly enriched in ribosomes, spliceosomes, ribosome biogenesis in eukaryotes, metabolic pathways, photosynthesis, and starch and sucrose metabolism (Figure 3F). Transcriptomic data analysis showed that overexpression of miR9748 might induce the expression of genes involved in ABA, ETH, and JA to participate in the response to high temperature stress.

### miR9748 Target Gene Identification

To further investigate the function mechanism of miR9748 under high temperature stress, we predicted the target gene of miR9748 using psRobot and Target Finder software. PsRobot predicted 467 target genes and Target Finder predicted 649 target genes, containing a total of 233 commonly regulated target genes. GO enrichment analysis was performed on 233 possible target genes. It was found that these target genes were successfully assigned to the corresponding 17 GO items. In molecular function category, 146 target genes were mainly enriched, among which 77 and 83 target genes were significantly enriched in catalytic activity (GO:0003824) and binding (GO:0005488), respectively. In cellular component group classification, 133 target genes were mainly enriched, and 97 and 76 target genes were significantly enriched in cell (GO:0005623) and organelle (GO:0043226), respectively. There were 126 target genes in biological processes, among which cellular processes (GO:0065007) and metabolic processes (GO:0008152) were significantly enriched, with 97 and 94 target genes, respectively (Supplementary Figure 2). Furthermore, four predicted target genes of cucumber miR9748 (CsaV3_6G007840, CsaV3_1G045520, CsaV3_7G029600, and CsaV3_5G039430) were related to plant hormone signal transduction, which was closely related to high temperature. In order to understand whether they are involved in high temperature stress, we detected their expression patterns under high temperature, and found that CsaV3_6G007840 was upregulated and then downregulated under high temperature treatment, and reached the peak at 2 h, with 5 times of that in 0 h (Supplementary Figure 3). The expression of CsaV3_1G045520 was downregulated at 1 h and then upregulated again, and reached the peak at 2 h after treatment, which was 1.8 times of that in 0 h (Supplementary Figure 3). The expression of CsaV3_7G029600 was similar with that of CsaV3_1G045520 (Supplementary Figure 3). However, the expression level of CsaV3_5G039430 was always downregulated (Supplementary Figure 3), which was opposite to the expression pattern of miR9748, suggesting that it might be the target gene of miR9748 under high temperature stress.

**Figure 4:**
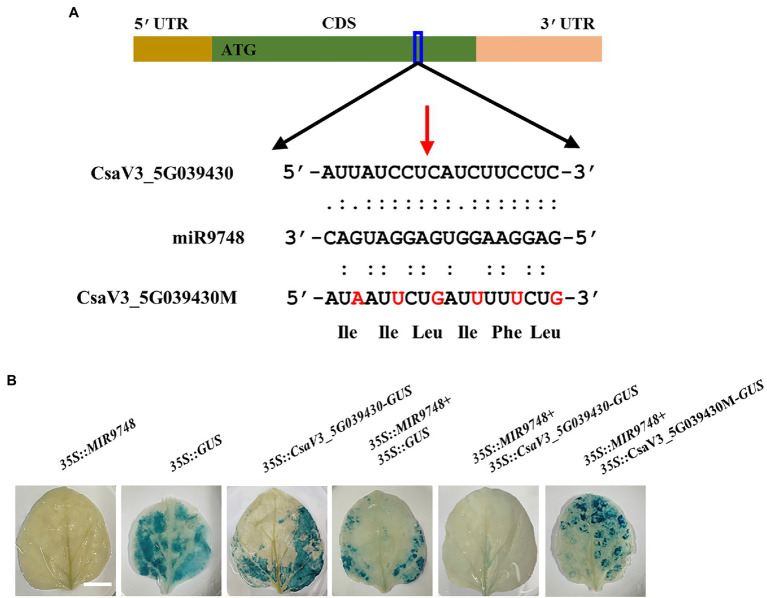
CsaV3_5G039430 is the target gene of miR9748. **(A)** 5′ RLM-RACE analysis of miR9748 cleavage site in CsaV3_5G039430, and the cleavage site is indicated by red arrow. **(B)** Transient GUS expression verification of miR9748 cleavage of CsaV3_5G039430. *Agrobacterium tumefaciens* containing the indicated plasmids were infiltrated into tobacco leaves, and the leaves were stained after infiltration for 2 d. Bar: 1 cm.

It is well known that plant miRNAs negatively regulate the expression of their target genes through either mRNA cleavage or translational inhibition (Zhao et al., 2016; Chen et al., 2018). To investigate whether CsaV3_5G039430 was the target gene of miR9748, we used 5′ RLM-RACE to locate miR9748-directed cleavage sites in CsaV3_5G039430. The results showed that there was a cleavage site between the 10th and 11th base pairs at the miR9748 target site (Figure 4A). To further verify whether CsaV3_5G039430 was the true target of miR9748, the interaction between miR9748 and CsaV3_5G039430 was verified using *Agrobacterium*-mediated transient expression in tobacco leaves. The results showed that when the transient transformation of *35S*::*MIR9748* overexpression vector, tobacco leaves were white after GUS staining and alcohol decolorization (Figure 4B). When *35S*::GUS or *35S*::CsaV3_5G039430-GUS or *35S*::*MIR9748* and *35S*::GUS were injected into tobacco leaves, GUS staining was found in tobacco leaves with blue color and large area, indicating that GUS was strongly expressed (Figure 4B). In contrast, when *35S*::*MIR9748* was co-transformed with *35S*::CsaV3_5G039430-GUS overexpression vector, the blue color became lighter and the area became smaller (Figure 4B). However, when *35S*::*MIR9748* was co-transformed with the mutated sites overexpression vector (*35S*::CsaV3_5G039430M-GUS), tobacco had large blue area and dark color (Figure 4B). These results indicated that CsaV3_5G039430 was the true target gene of miR9748, and miR9748 negatively regulated the expression of CsaV3_5G039430 through cleavage.

### Functional Analysis of CsaV3_5G039430 Under High Temperature Stress

To further investigate the role of CsaV3_5G039430 under high temperature stress, we searched this gene in cucurbitaceae genome database and found that it was a NRT1/PTR family 4.4 protein (Supplementary Table 5), and an orthologous of AtNRT1.13/NPF4.4, which is lack of the conserved proline residue between the 10^th^ and 11^th^ transmembrane domains, resulting in only binding nitrate and without transport activity (Chen et al., 2021a). Therefore, the protein encoded by CsaV3_5G039430 was named as CsNPF4.4, which shared 63.30% sequence identity and had the conserved domains and similar molecular weight and theoretical isoelectric point (pI) with AtNPF4.4 (Supplementary Figure 4; Supplementary Table 5). Tissue expression analysis showed that *CsNPF4.4* had lower expression in flowers, fruits, and leaves and higher expression in roots and stems (Supplementary Figure 5A). The expression level of *CsNPF4.4* in roots was approximately 5-fold that in leaves and 2.5-fold that in stems (Supplementary Figure 5A), indicating that *CsNPF4.4* was predominantly expressed in cucumber roots. Subcellular localization results showed that tobacco cells transformed with *35S*::*CsNPF4.4*-GFP detected green fluorescence both in cell membrane, cytosol, and nucleus (Supplementary Figure 5B).

**Figure 5:**
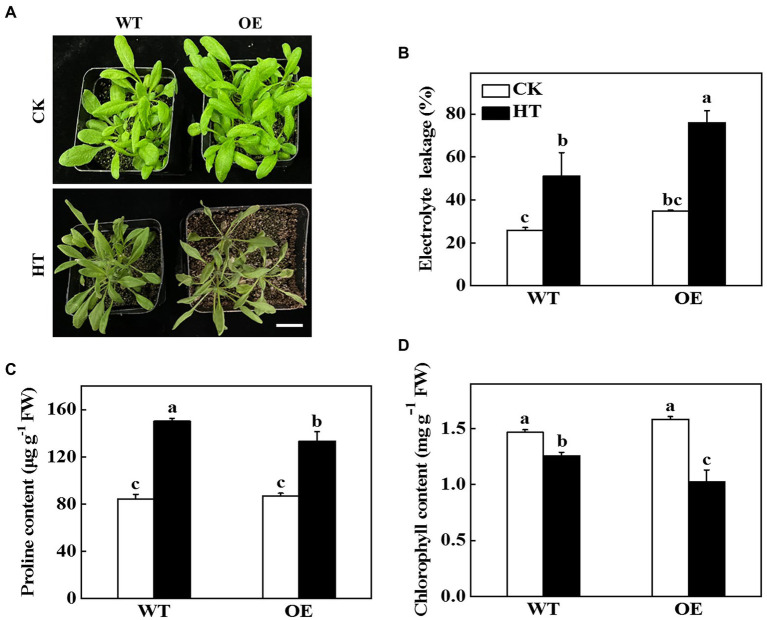
Functional analysis of *CsNPF4.4* in response to high temperature stress. **(A)** Overexpression of cucumber *CsNPF4.4* in Arabidopsis reduced high temperature stress tolerance. Bar: 1 cm. **(B)** Electrolyte leakage. **(C)** Proline content in leaves. **(D)** Chlorophyll content. 35-d-old Arabidopsis seedlings were subjected to high temperature stress for 2 d, and the phenotype, electrolyte leakage, proline, and chlorophyll content were measured. The results represent the mean ± SD (*n* = 3). Means with the same letter did not significantly differ at *p* < 0.05 according to Tukey’s test. CK, control; HT, high temperature; FW, fresh weight.

To further test the role of *CsNPF4.4* under high temperature stress, the *35S*:: *CsNPF4.4* overexpression vector was constructed and transformed into Arabidopsis. The expression level of *CsNPF4.4* in overexpression lines (OE) was 18-fold higher than that in WT plants (Supplementary Figure 6A). Before high temperature treatment, OE plants had more and larger leaves than WT plants (Figure 5A; Supplementary Figure 6B). After 2 d of high temperature treatment, OE plants were more sensitive to high temperature than WT plants (Figure 5A). The fresh weight of WT and OE plants decreased by 19.34 and 27.51%, and the dry weight decreased by 15.87 and 23.87%, respectively, compared with their own control plants (Supplementary Figures 6B,C). High temperature stress increased the value of electrolyte leakage of WT and OE plants, as indicated increased by 28.2% in WT plants and 34.7% in OE plants (Figure 5B). Furthermore, high temperature promoted the accumulation of proline, but the proline content in OE plants was still lower than that in WT plants (Figure 5C). Although high temperature stress reduced chlorophyll content both in WT and OE plants, the chlorophyll content in WT plants was significantly higher than that in OE plants (Figure 5D). These results indicated that *CsNPF4.4* overexpression plants suffered more damage under high temperature treatment, and had the opposite effect of miR9748.

### CsNPF4.4 Negatively Regulates High Temperature Stress Tolerance Through Inhibition JA

**Figure 6:**
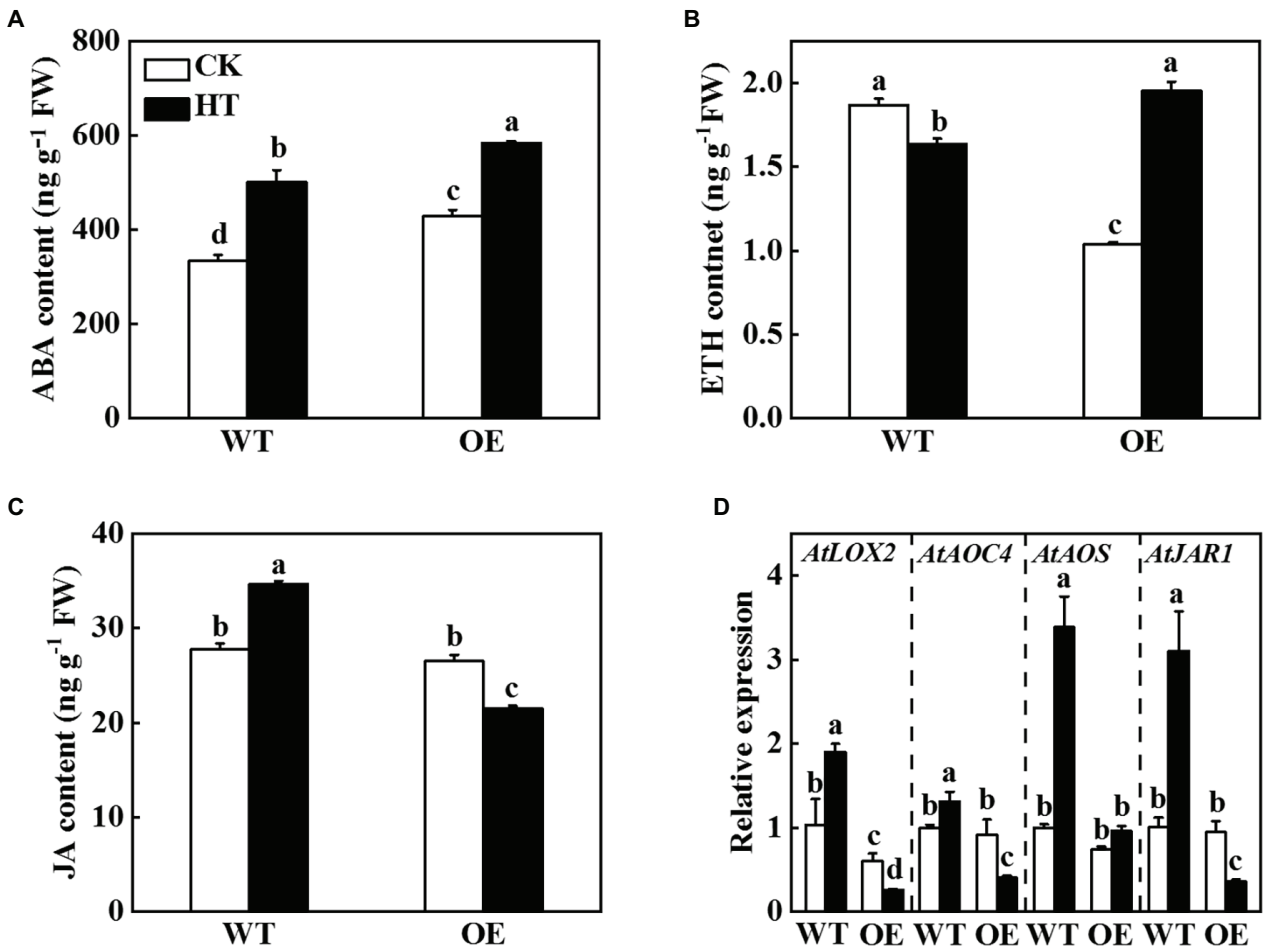
Effects of *CsNPF4.4* on plant hormone content and the expression of genes related to jasmonic acid (JA) synthesis under high temperature stress. **(A)** Abscisic acid content. **(B)** Ethylene content. **(C)** Jasmonic acid content. 35-d-old Arabidopsis seedlings were subjected to high temperature stress for 2 d, and the plant hormone contents were measured. **(D)** Effects of *CsNPF4.4* on the expression of genes related to JA synthesis under high temperature. The leaf samples were harvested after high temperature stress for 24 h and analyzed by qPCR. The results represent the mean ± SD (*n* = 3). Means with the same letter did not significantly differ at *p* < 0.05 according to Tukey’s test. CK, control; HT, high temperature; FW, fresh weight.

The transcriptome sequencing results of Arabidopsis suggested that overexpression of miR9748 regulated the expression of genes related to ABA, ETH, and JA (Figure 3). *CsNPF4.4* acted as the target gene of miR9748, it might regulate plant hormones to mediate high temperature stress tolerance. Therefore, we measured the content of ABA, ETH, and JA in WT and OE plants. The ABA and ETH contents in OE plants were significantly higher than those of WT plants under high temperature stress (Figures 6A,B). High temperature stress induced the accumulation of JA in WT plants, but decreased in OE plants, as indicated by the content of JA significantly lower than that of WT plants (Figure 6C). Furthermore, high temperature induced the expression of JA synthesis genes, such as *LOX2*, *AOC4*, *AOS*, and *JAR1*, in WT plants (Figure 6D). However, the expression level of *LOX2*, *AOC4*, and *JAR1* significantly decreased in OE plants under high temperature stress compared with the control plants (Figure 6D). Thus, *CsNPF4.4* might negatively regulate high temperature tolerance by downregulating the expression of genes involved in JA synthesis and reducing JA content.

**Figure 7:**
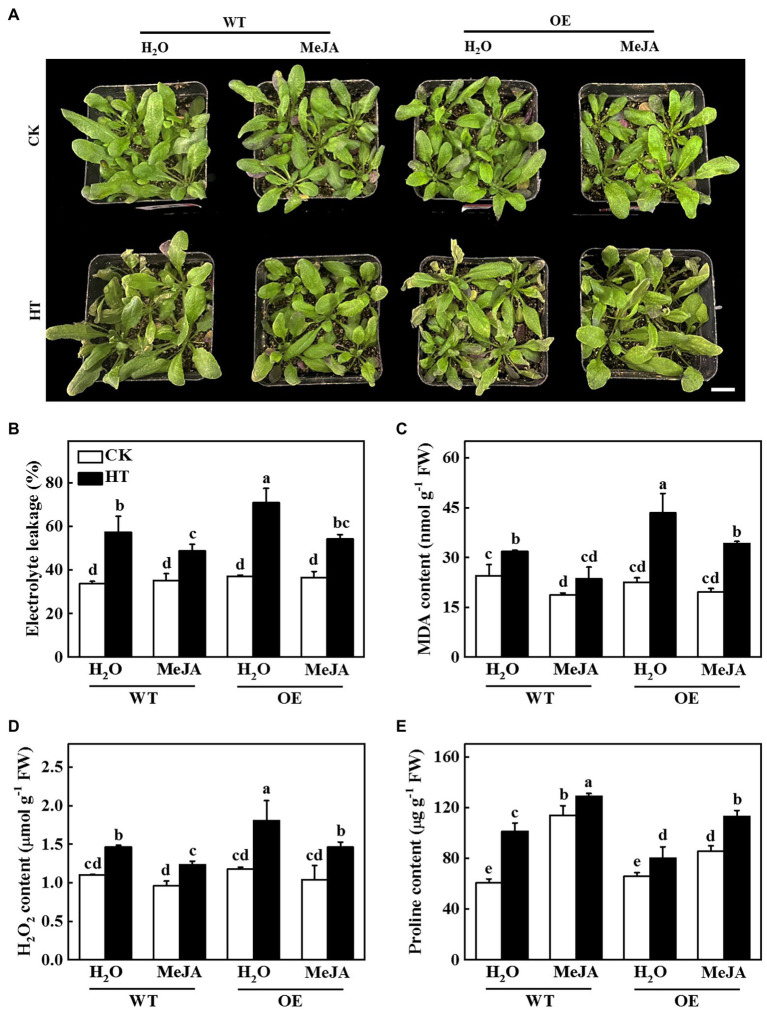
Functional analysis of methyl jasmonate (MeJA) on *CsNPF4.4* overexpression plants in response to high temperature stress. **(A)** Exogenous spraying of MeJA alleviated the damage of high temperature to *CsNPF4.4* overexpression plants. Bar: 1 cm. **(B)** Electrolyte leakage. **(C)** Malondialdehyde (MDA) content. **(D)** H_2_O_2_ content. **(E)** Proline content. 35-d-old Arabidopsis seedlings were subjected to high temperature stress for 2 d, and the phenotype, electrolyte leakage, MDA, and proline content were measured. The results represent the mean ± SD of 3 replicates. Means with the same letter did not significantly differ at *p* < 0.05 according to Tukey’s test. CK, control; HT, high temperature; FW, fresh weight.

To investigate whether JA was involved in *CsNPF4.4*-mediated response to high temperature stress, we sprayed WT and OE plants with 100 μmol MeJA and then analyzed their high temperature stress tolerance. After 2 d of high temperature stress, WT plants-treated with distilled water showed wilting and coking of leaf margins, but the OE plants were more serious (Figure 7A). However, the WT and OE plants were alleviated after spraying MeJA (Figure 7A). Furthermore, sprayed with MeJA inhibited high temperature-induced the increase of electrolyte leakage, MDA and H_2_O_2_ content, and also increased proline content both in WT and OE plants compared with the distilled water-treated plants (Figures 7B–E), indicating that exogenous spraying of MeJA could ameliorate the high temperature sensitivity of *CsNPF4.4* overexpression plants.

### CsbZIP2 Binds to the Promoter of *MIR9748* to Promote Its Transcription

**Figure 8:**
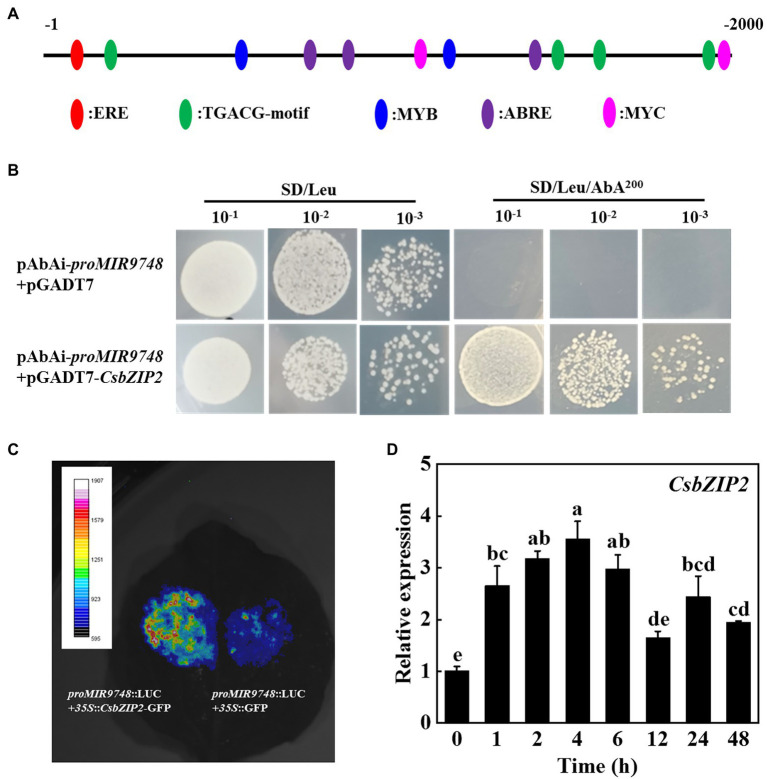
CsbZIP2 binds to the promoter of *MIR9748* and high temperature stress induces the expression of *CsbZIP2*. **(A)** Analysis of cis-acting elements in *MIR9748* promoter. Numbering is from predicted transcriptional start sites. **(B)** Yeast one-hybrid assay showing the binding of CsbZIP2 to *MIR9748* promoter. Yeast cells with DNA–protein interactions were grown on SD/Leu plates with 200 ng ml^−1^ aureobasidin A. **(C)** Luciferase assay showing transient overexpression of *CsbZIP2* enhancement the expression of *MIR9748* in *Nicotiana benthamiana* leaves. The empty vector was used as a control. **(D)** qPCR analysis the expression level of *CsbZIP2* under high temperature stress. The leaf samples were harvested at the indicated time points and analyzed by qPCR. The results represent the mean ± SD (*n* = 3). Means with the same letter did not significantly differ at *p* < 0.05 according to Tukey’s test.

In order to identify the transcription factors that regulate the expression of *MIR9748* gene, we first used the online software PlantCARE to analyze the *MIR9748* promoter sequence, and found that there was multiple ABA response element (ABRE), JA response elements (TGACG-motif, TGACG-motif, CGTCA-motif) and a variety of light-responsive elements (G-box, GATA-motif), and MYB, MYC, ERE, and other functional elements (Figure 8A).

We used yeast one-hybrid experiments to screen the transcription factors that bound to the promoter of *MIR9748*. The results showed that yeast cells containing the bait vector harboring *MIR9748* promoter sequence grew on the SD/Leu medium containing 200 ng ml^−1^ AbA when transformed with pGADT7-CsbZIP2, but those transformed with pGADT7 vector or other transcription factors could not grow on the same selection medium (Figure 8B; Supplementary Figure 7), indicating that CsbZIP2 bound to the promoter of *MIR9748 in vitro*. To further validate this results, we performed dual-luciferase assay using the tobacco transient transformation system. As shown in Figure 8C, the fluorescence signal was stronger when co-injected with *proMIR9748*-LUC and *35S*::*CsbZIP2* than that co-injected with *proMIR9748*-LUC and *35S*::GFP. Furthermore, the transcription level of *CsbZIP2* was significantly increased under high temperature treatment, and reached the maximum value at 4 h of high temperature stress, which increased by 2.5-fold compared with that of 0 h (Figure 8D), indicating that CsbZIP2 responded to high temperature stress. Thus, the transcription factor CsbZIP2 was upregulated under high temperature stress to promote the expression of *MIR9748* gene in cucumber leaves to negatively regulate the expression of *CsNPF4.4*, resulting in improving high temperature tolerance.

## Discussion

It has been demonstrated that miR9748 is involved in the regulation of plant growth and development and abiotic stress response (Cakir et al., 2016; Sun et al., 2017; Cheng et al., 2019). Tomato miR9748 might regulate the expression of *ERD* (early response to dehydration-like), *DREB* (dehydration response element binding), and *DI19* (dehydration/drought-induced 19 protein) in response to drought stress (Candar-Cakir et al., 2016). Cowpea (*Vigna unguiculata* L.) miR9748 targets heat shock proteins expression in response to heat stress (Gul et al., 2017). Selenium treatment induced the expression of miR9748 in *A. chrysochlorus* to further regulate the expression of *MYC2* and *HSP90* (Cakir et al., 2016). Here, we found that cucumber miR9748 was significantly upregulated under high temperature treatment (Figure 1B), indicating that miR9748 might play a critical role in high temperature. Indeed, overexpression of cucumber miR9748 in Arabidopsis enhanced the resistance to high temperature stress compared with WT plants, as indicated by miR9748 overexpression plants maintaining higher level of proline and chlorophyll content, and lower H_2_O_2_ content and electrolyte leakage (Figure 2). Our previous study revealed that miR9748 may interact with lncRNAs and circRNAs to mediate high temperature stress through plant hormone signal transduction pathways (He et al., 2020). In the present study, transcriptome data showed that the DEGs were mainly related to the response to ABA, ETH, and JA (Figure 3), revealing that miR9748 might increase high temperature stress tolerance *via* regulating these plant hormones signal pathways.

Plant miRNAs mediate growth and development, and response to various stresses through negative regulation target genes expression or translation (Chen et al., 2018; Bhogireddy et al., 2021). miR1432 negatively regulates the expression of target gene *OsACOT* (Acyl-CoA thioesterase) and increases grain filling amount to regulate rice yield (Zhao et al., 2019). Defense against rice streak virus invasion leads to a reduction of miR528 in rice, alleviating miR528-mediated degradation of L-ascorbate oxidase mRNA, and enhancing rice antiviral activity (Yao et al., 2019). In addition, numerous of miRNAs are identified in the regulation of high temperature response in plants (Sailaja et al., 2014; Mangrauthia et al., 2017; Ravichandran et al., 2019). ABA inhibits the expression of miR159b, which downregulates the expression of its target genes, such as *GAMYB1*, *MYB29*-like, and *HSP70*, to enhance high temperature tolerance in grafted cucumber plants (Li et al., 2016). Heat stress induces the expression of miR4200 to degrade the mRNA of *HSFB4a*, a negative regulator in heat stress, thereby exhibiting higher heat stress tolerance in tomato (Rao et al., 2022). Similarly, HSFA1b and HSFA7b induce miR398 to increase thermotolerance of Arabidopsis through downregulation the expression of *CSD1*, *CSD2,* and *CCS* (Guan et al., 2013). Furthermore, miR156*-SPL* module regulates the response to recurring heat stress in Arabidopsis (Cui et al., 2014; Sailaja et al., 2014). In this study, it was demonstrated that the expression of miR9748 and *CsNPF4.4* was upregulated and downregulated, respectively, under high temperature stress (Figure 1B; Supplementary Figure 3). 5′ RLM-RACE technology and tobacco transient co-transformation experiments found that *CsNPF4.4* was a target gene of miR9748 in cucumber (Figure 4). Our results suggested that miR9748 was involved in the response to high temperature by precisely cleaving *CsNPF4.4*. CsNPF4.4 is a possible peptide/nitrate transporter involved in peptide/nitrate transport. Studies have shown that NRT1.1 in Arabidopsis regulates NO_3_^−^ distribution to roots by coordinating the accumulation of Cd^2+^ in root vacuoles, thereby promoting Cd^2+^ detoxification (Jian et al., 2019). Furthermore, NRT1.1 suppresses lateral root development through inhibiting the expression of auxin synthesis and auxin influx carrier genes and promoting basipetal auxin transport out of the lateral root primordia at low-nitrate availability in Arabidopsis (Krouk et al., 2010; Zhang et al., 2019; Maghiaoui et al., 2020). In *Medicago truncatula*, NPF6.8 mediates high nitrate-induced repression of primary root growth *via* ABA (Pellizzaro et al., 2014). Moreover, *nrt2* mutants increase the tolerance to *Pseudomonas syringae* pv *tomato* DC3000 through inducing the accumulation of salicylic acid (Camanes et al., 2012). These results suggest that NRT not only regulates the uptake and transport of nitrate, but also mediates the homeostasis of plant hormones to response to environmental stresses. AtNPF4.4, an orthologous of CsNPF4.4, lacks of the conserved proline between the 10th and 11th transmembrane domain and cannot transport nitrate but can bind it (Chen et al., 2021a). Deficient of *AtNPF4.4* exhibits delayed flowering, enhanced node number, inhibited branch outgrowth, and lateral nitrate allocation to nodes under low-nitrate conditions (Chen et al., 2021a). Amino acid sequence alignment showed that CsNPF4.4 also did not contain this conserved proline (Supplementary Figure 4), indicating that CsNPF4.4 might have the similar function in nitrate transport. Interestingly, overexpression of *CsNPF4.4* promoted plants growth under the normal growth conditions, as indicated by higher fresh and dry weight compared with WT plants (Supplementary Figures 6B,C). However, their roles in high temperature response are largely unknown. Here, we found that *CsNPF4.4* overexpression plants were more sensitive to high temperature stress accompanied by inhibition JA accumulation (Figures 5, 6C). It has been shown that NRT displays transport plant hormones, including auxin, ABA, gibberellin, and jasmonoyl-isoleucine (Chiba et al., 2015). Interestingly, low nitrogen stress enhances lateral root number and JA content in wheat (*Triticum aestivum* L.), while high nitrate inhibits root growth in maize (*Zea mays* L.) and decreases JA content (Saiz-Fernández et al., 2020; Lv et al., 2021). Here, we found that JA content in *CsNPF4.4* overexpression plants was no significant difference with that in WT plants under normal growth conditions (Figure 6C). However, the expression of JA synthesis genes and JA content in *CsNPF4.4* overexpression plants dramatically decreased under high temperature stress (Figures 6C,D). These results indicate that NRT is involved in JA synthesis under adverse conditions, but the specific functional mechanism remains further investigation.

It has been demonstrated that JA is involved in the regulation of plant response to high temperature stress (Xia et al., 2015; Balfagon et al., 2019). Exogenous spraying of MeJA improves high temperature tolerance of plants through regulation osmotic adjustment, antioxidant defense, maintenance the stability of photosynthesis proteins, and inducing the expression of JA-responsive genes (Fatma et al., 2021; Su et al., 2021). Silencing of *WRKY6* increases the susceptibility to heat stress in pepper by downregulation of JA-induced gene expression (Cai et al., 2015). HsfA1b mediates heat resistance *via* OPR3 and JA signal pathway in wheat and Arabidopsis (Tian et al., 2020). High temperature stress significantly induces the expression of JA pathway genes and the production of JA, thereby improving the adaptability to heat stress (Su et al., 2021). Nevertheless, *CsNPF4.4* overexpression plants compromised the expression of JA synthesis genes and the increase of JA under high temperature stress (Figures 6C,D), resulting in reduced the tolerance to high temperature. Exogenous application of MeJA significantly alleviated the damage of high temperature to *CsNPF4.4* overexpression plants (Figure 7). Therefore, miR9748 directly cleaved the target gene *CsNPF4.4* to increase the content of JA, resulting in enhancing the tolerance to high temperature stress.

Plant bZIP transcription factors play an indispensable role in the regulation of plant high temperature stress response (Droege-Laser et al., 2018). Overexpression of *OsbZIP46* in rice and overexpression of wheat *TabZIP60* in Arabidopsis improve the high temperature tolerance of transgenic plants (Chang et al., 2017; Geng et al., 2018). Knockdown the expression of *bZIP60* in maize increases the hypersensitivity to high temperature stress and compromised high temperature-induced the expression of genes related to *HSFs*, chlorophyll metabolism and chloroplast protein turnover (Li et al., 2020). Furthermore, maize *bZIP4* is induced by high temperature stress (Ma et al., 2018). Similarly, *CsbZIP2* was induced by high temperature, and the transcription level of *CsbZIP2* was significantly increased under high temperature (Figure 8D). Interestingly, yeast one-hybrid and dual-luciferase reporter assays found that the transcription factor CsbZIP2 could bind to the promoter of *MIR9748* to promote its transcription (Figure 8).

**Figure 9:**
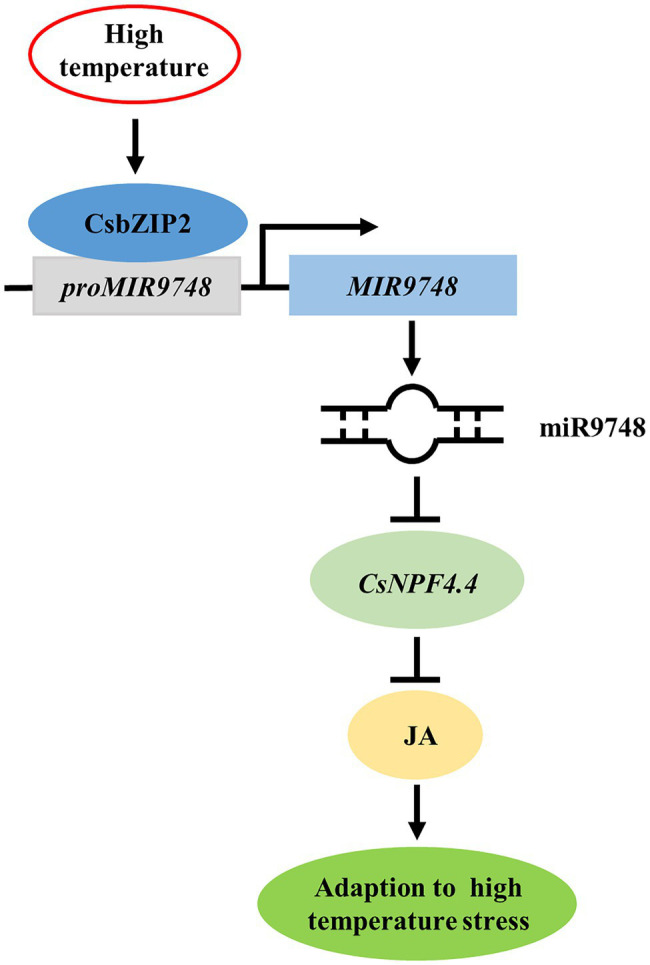
Proposed model of CsbZIP2-miR9748-CsNPF4.4 module in response to high temperature stress. High temperature induced the expression of CsbZIP2, which bound to the promoter of *MIR9748* to induce its transcription to form mature miR9748. miR9748 negatively regulated target gene *CsNPF4.4* through direct cleavage, to promote the accumulation of JA, resulting in enhancing high temperature tolerance.

In conclusion, high temperature stress induced the expression of CsbZIP2, which bound to the promoter of *MIR9748* to promote its transcription to form mature miR9748. miR9748 negatively regulated target gene *CsNPF4.4* through direct cleavage. Overexpression of *CsNPF4.4* decreased high temperature tolerance and subdued high temperature-induced the increase of JA, while foliar application of MeJA mitigated the sensitivity of *CsNPF4.4* overexpression plants to high temperature stress. Thus, high temperature stress induced CsbZIP2 to trigger the expression of miR9748, which negatively regulated target gene *CsNPF4.4* through direct cleavage, to promote the accumulation of JA, resulting in enhancing high temperature tolerance (Figure 9). Our results provide a new perspective for elucidating the response mechanism of cucumber to high temperature stress.

## Data Availability Statement

The datasets presented in this study can be found in online repositories. The names of the repository/repositories and accession number(s) can be found in the article/Supplementary Material.

## Author Contributions

JS and YW designed the experiment. LL, GC, and MY performed the experiments. LL and SG analyzed the data. LL and YW wrote the manuscript. JS revised the manuscript. All authors contributed to the article and approved the submitted version.

## Funding

This work was supported by the National Key Research and Development Program of China (2019YFD1001900), the China Agriculture Research System (CARS-23), and the Postdoctoral Research Funding Scheme of Jiangsu Province (2019K071).

## Conflict of Interest

The authors declare that the research was conducted in the absence of any commercial or financial relationships that could be construed as a potential conflict of interest.

## Publisher’s Note

All claims expressed in this article are solely those of the authors and do not necessarily represent those of their affiliated organizations, or those of the publisher, the editors and the reviewers. Any product that may be evaluated in this article, or claim that may be made by its manufacturer, is not guaranteed or endorsed by the publisher.
