# Supplementary material for: CsbZIP2-miR9748-CsNPF4.4 Module Mediates High Temperature Tolerance of Cucumber Through Jasmonic Acid Pathway
Source: Front Plant Sci. 2022 Apr 28;13:883876. doi: 10.3389/fpls.2022.883876 (PMC9096661; doi:10.3389/fpls.2022.883876)
Supplement: Supplementary file 1 [file Data_Sheet_1.pdf]

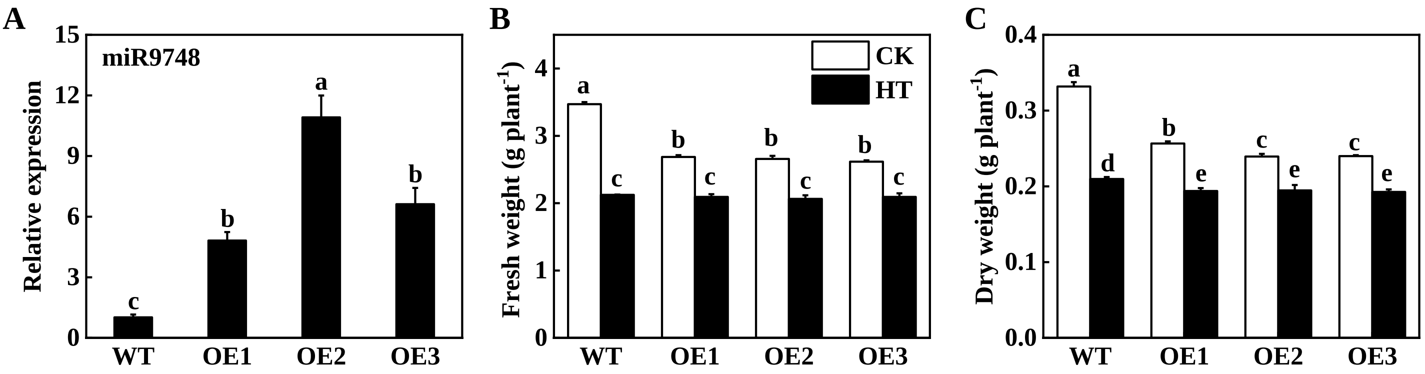

SUPPLEMENTARY FIGURE 1. qPCR analysis the expression of miR9748 in overexpression plants and effects of high temperature stress on biomass of miR9748 overexpression plants. (**A**) Relative expression level of miR9748 in transgenic Arabidopsis plants. (**B**) Fresh weight. (**C**) Dry weight. 35-d-old Arabidopsis seedlings were subjected to high temperature stress for 2 d, and the fresh and dry weight were measured. The results represent the mean ± SD of 3 replicates. Means with the same letter did not significantly differ at $p < 0.05$ according to Tukey's test. CK, control; HT, high temperature.

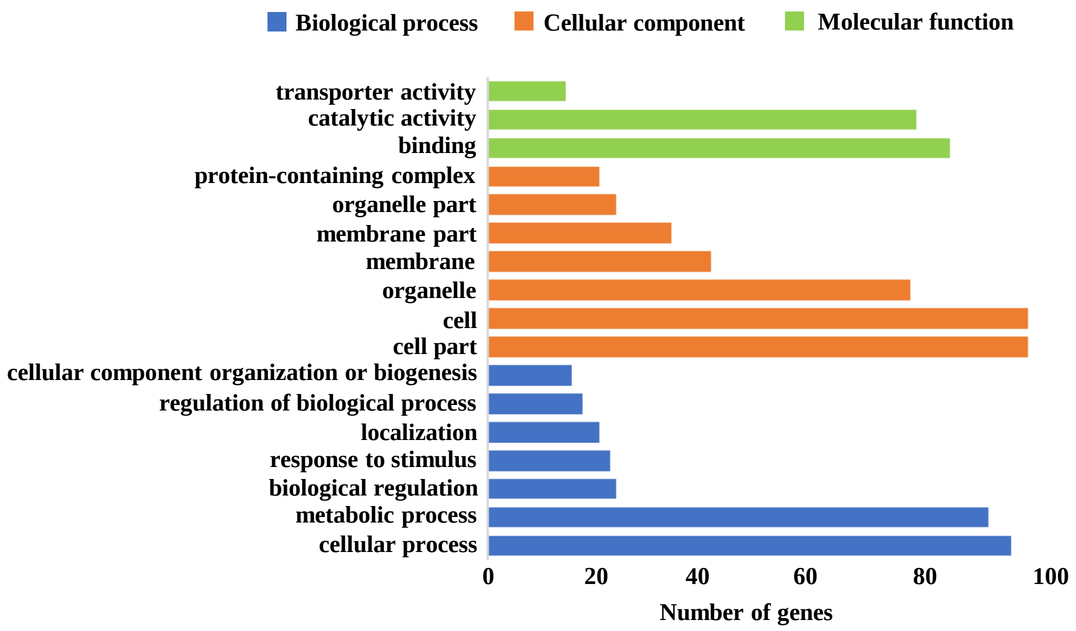

SUPPLEMENTARY FIGURE 2. GO enrichment analysis of miR9748 target genes.

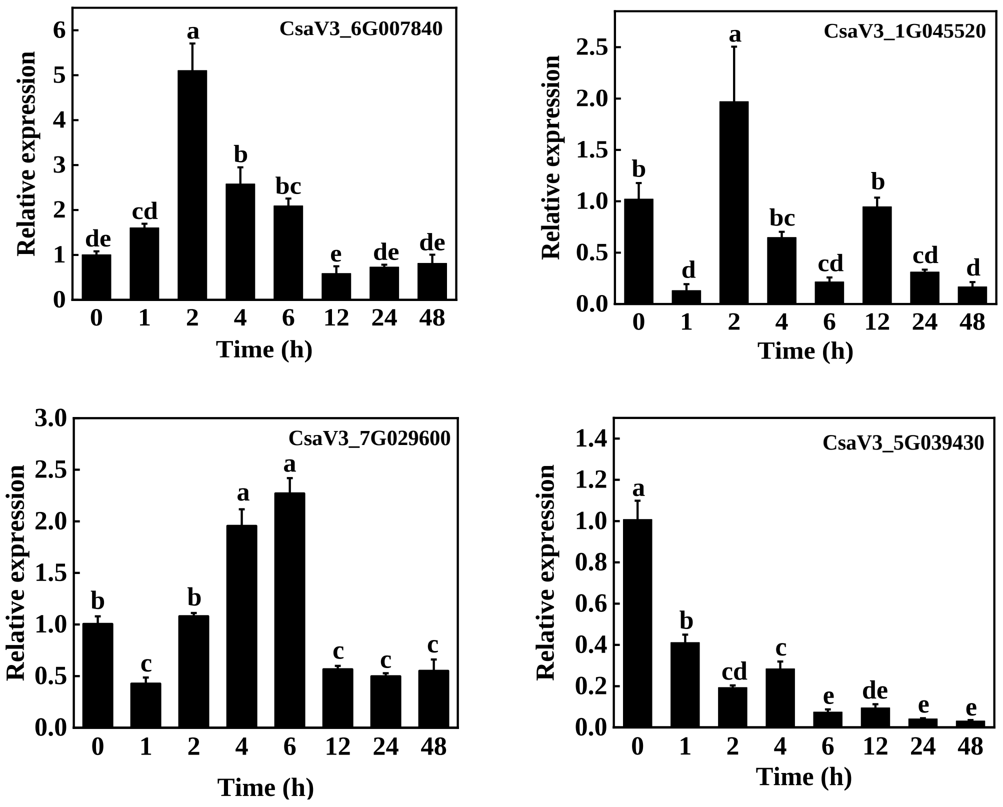

SUPPLEMENTARY FIGURE 3. Expression patterns of predicted miR9748 target genes in cucumber under high temperature stress. qPCR analysis the expression level of predicted miR9748 target genes under high temperature stress. The leaf samples were harvested at the indicated time points and analyzed by qPCR. The results represent the mean ± SD of 3 replicates. Means with the same letter did not significantly differ at $p < 0.05$ according to Tukey's test.

```
AtNRT1.13/NPF4.4  MDVHDLSEEAKRGVIHTSEESLDDLCVDFRGRPCRPSKHGGTRAALFVLGFQAFEMMAIA  60
CsNPF4.4          -----------------MQRDHEDICVDWRGRACNPNRHGGMGAAAFVLGLQAFEMMAIA  43
AtCHL1/NPF6.3     -----------MSLPETKSDDILLDAWDFQGRPADRSKTGGWASAAMILCIEAVERLTTL  49

AtNRT1.13/NPF4.4  AVGNNLITYVFNEMHFPLSKSANLVTNFIGTVFLLSLLGGFLSDSYLGSFRTMLVFGVIE  120
CsNPF4.4          AVGNNLITYVFSEMHFPLSKSANVVTNFVGTVFFLSLLGGFLSDSYLGSFRTMLIFGFIE  103
AtCHL1/NPF6.3     GIGVNLVTYLTGTMHLGNATAANTVTNFLGTSFMLCLLGGFIADTFLGRYLTIAIFAAIQ  109

AtNRT1.13/NPF4.4  ISGFILLSVQAHLPELRPPECNMKSTT----IHCVEANGYKAATLYTALCLVALGSGCLK  176
CsNPF4.4          LAGFTVLTIQAHYPGLRPSPCEMGRKLDEEDDQCIEAKGYEALMFFSALYLVALGSGCLK  163
AtCHL1/NPF6.3     ATGVSILTLSTIIPGLRPPRCNPTTSS-----HCEQASGIQLTVLYLALYLTALGTGGVK  164

AtNRT1.13/NPF4.4  PNIISHGANQFQRKD---LRKLSSFFNAAYFAFSMGQLIALTLLVWVQTHSGMDVGFGVS  233
CsNPF4.4          PNIISHGADQFRKEDPKQFKKLSTFFNCAYFAFCTGELIALTLLVWVQTHSGMDLGFGVS  223
AtCHL1/NPF6.3     ASVSGFGSDQFDETEPKERSKMTYFFNRFFFCINVGSLLAVTVLVYVQDDVGRKWGYGIC  224

AtNRT1.13/NPF4.4  AAVMAAGMISLVAGTSFYRNKPPSGSIFTPIAQVFVAAITKR-KQICPS-----NPNMVH  287
CsNPF4.4          TAAMLLALISLLSGTSFYRNNPPRGSIFTPIAQVLVAAYRKRNLQMCPS-----NSEMVL  278
AtCHL1/NPF6.3     AFAIVLALSVFLAGTNRYRFKKLIGSPMTQVAAVIVAAWRNRKLELPADPSYLYDVDDII  284

AtNRT1.13/NPF4.4  QPSTDLVRVKPLLHSNKFRFLDKACIKTQG-----KAMESPWRLCTIEQVHQVKILLSVI  342
CsNPF4.4          NG--TQNGIVKLIHTDKFRFLDKACIKSEEERLGSIREESPWKLCTVKQVEQVKIILSVI  336
AtCHL1/NPF6.3     AAEGSMKGKQKLPHTEQFRSLDKAAIRDQEAG-VTSNVFNKWTLSTLTDVEEVKQIVRML  343

AtNRT1.13/NPF4.4  PIFACTIIFNTILAQLQTFSVQQGSSMNTHITKTFQIPPASLQAIPYIILIFFVPLYETF  402
CsNPF4.4          PIFACTIIFNTILAQLQTFSVQQGASMNTWLTNSFQIPPASLQAIPYIILIFLVPLYETV  396
AtCHL1/NPF6.3     PIWATCILFWTVHAQLTTLSVAQSETLDRSIG-SFEIPPASMAVFYVGGLLLTTAVYDRV  402

AtNRT1.13/NPF4.4  FVPLARKLTGNDSGISPLQRIGTGLFLATFSMVAAALVEKKRRESFLE-----QNVMLSI  457
CsNPF4.4          FVPLTRRLTSIDSGISPLQRVGTGLFVATFSMVSAALVEQKRRN---------SSSSLSI  447
AtCHL1/NPF6.3     AIRLCKKLFNYPHGLRPLQRIGLGLFFGSMAMAVAALVELKRLRTAHAHGPTVKTLPLGF  462
                     TMH10                   *             TMH11
AtNRT1.13/NPF4.4  FWIAPQFLIFGLSEMFTAVGLVEFFYKQSSQSMQSFLTAMTYCSYSFGFYLSSVLVSTVN  517
CsNPF4.4          LWIAPQFVIFGVSELFTAVGLIEFFYKQSVEGMQSFLTAMTYCSYSFGFYLSSLLVSLVN  507
AtCHL1/NPF6.3     YLLIPQYLIVGIGEALIYTGQLDFFLRECPKGMKGMSTGLLLSTLALGFFFSSVLVTIVE  522

AtNRT1.13/NPF4.4  RVTSSNGSGTKEGWLGDNDLNKDRLDHFYWLLASLSFINFFNYLFWSRWYSCDPS-ATHH  576
CsNPF4.4          KISG--------GWLSHNDLNNDRLDLFYWLLAGLSFVNFFNYLFWANRFSQQPP-LPLH  558
AtCHL1/NPF6.3     KFTG-----KAHPWIAD-DLNKGRLYNFYWLVAVLVALNFLIFLVFSKWYVYKEKRLAEV  576

AtNRT1.13/NPF4.4  SAEVNSLEALENGEIKDSTTEKPRI                                    601
CsNPF4.4          LLQTQTSTKQPSNSKLVVP------                                    577
AtCHL1/NPF6.3     GIELDDEPSIPMGH-----------                                    590
```

SUPPLEMENTARY FIGURE 4. Amino acid sequence alignment of NPF4.4 in Arabidopsis and cucumber. The amino acid alignment was performed using the Bioxm2.7 software. The red star indicates the conserved proline 492 in AtCHL1/NPF6.3 between the 10th and 11th transmembrane helix regions (TMH).

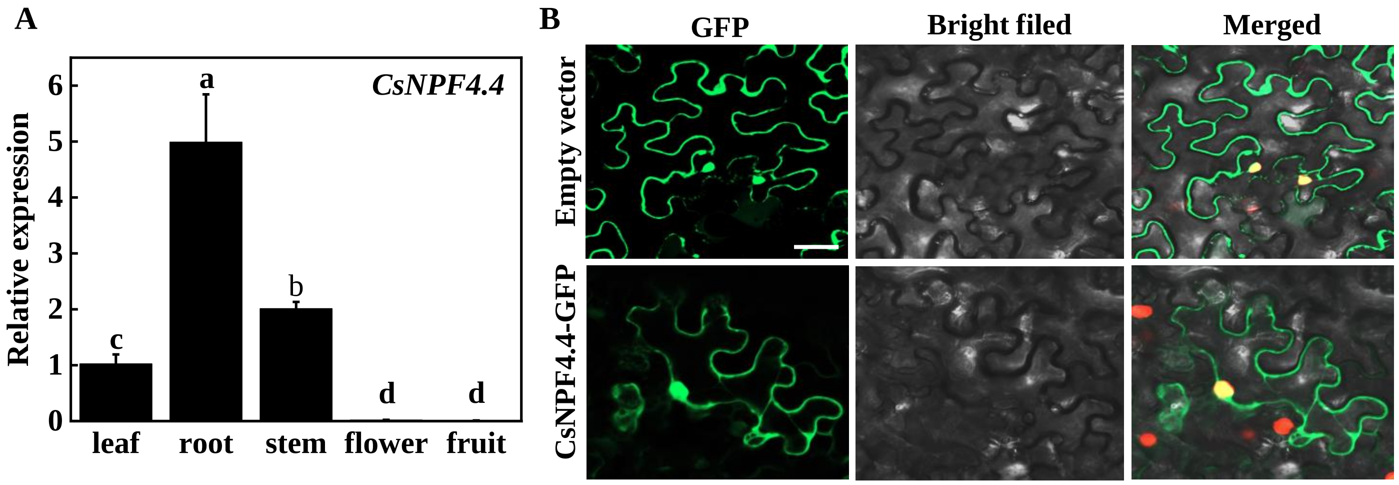

SUPPLEMENTARY FIGURE 5. Expression patterns of *CsNPF4.4* in different tissues and its subcellular localization. (**A**) qPCR analysis the expression of *CsNPF4.4* in leaves, roots, stems, flowers, and fruits of cucumber. The expression level in leaves was set to 1.0. The results represent the mean ± SD of 3 replicates. Means with the same letter did not significantly differ at $p < 0.05$ according to Tukey's test. (**B**) Subcellular localization of CsNPF4.4 in tobacco leaves. The GFP and RFP (a marker for nuclear localization) signals were visualized using confocal microscopy after infiltration for 48 h. Bar: 50 µm.

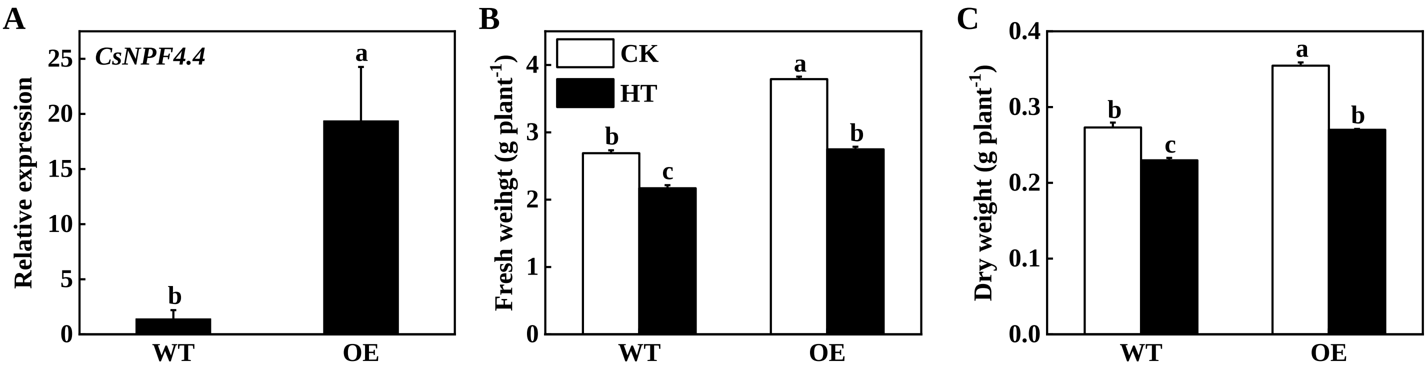

SUPPLEMENTARY FIGURE 6. qPCR analysis the expression of *CsNPF4.4* in overexpression plants and effects of high temperature stress on biomass of *CsNPF4.4* overexpression plants. (**A**) Relative expression level of *CsNPF4.4* in transgenic Arabidopsis plants. (**B**) Fresh weight. (**C**) Dry weight. 35-d-old Arabidopsis seedlings were subjected to high temperature stress for 2 d, and the fresh and dry weight were measured. The results represent the mean ± SD of 3 replicates. Means with the same letter did not significantly differ at $p < 0.05$ according to Tukey's test. CK, control; HT, high temperature.

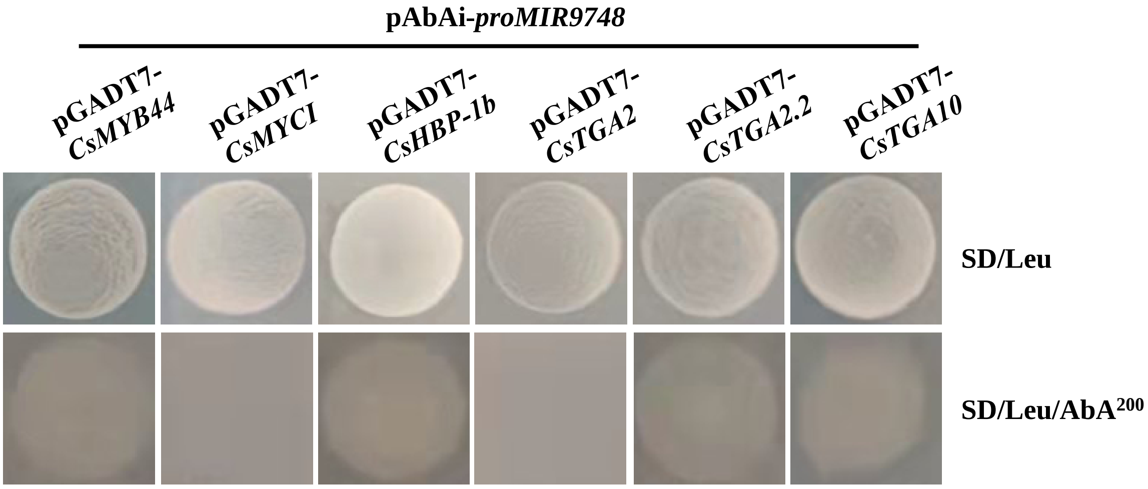

SUPPLEMENTARY FIGURE 7. Yeast one-hybrid assay screening the transcription factors binding to *MIR9748* promoter. Yeast cells harboring the indicated plasmids were grown on the selection medium.
