# Supplementary material for: CsbZIP2-miR9748-CsNPF4.4 Module Mediates High Temperature Tolerance of Cucumber Through Jasmonic Acid Pathway
Source: Front Plant Sci. 2022 Apr 28;13:883876. doi: 10.3389/fpls.2022.883876 (PMC9096661; doi:10.3389/fpls.2022.883876)
Supplement: Supplementary file 2 [file Data_Sheet_2.PDF]

Supplementary Table 1. Primer sequences for vector construction.

Primer name	Forward primer	Reverse primer
pFGC1008-*CsNPF4.4*	5'-ttacaattaccatggggcgcgccATGCAAAGAGATCATGAAGATATTTGT-3'	5'-agttaattaagctgaggatccTCAAGGCACTACAAGCTTAGAGTTTG-3'
pBI121-*CsaV3_5G039430*	5'-gagaacacgggggactctagaATGCAAAGAGATCATGAAGATATTTGT-3'	5'-ataagggactgaccacccgggTCAAGGCACTACAAGCTTAGAGTTTG-3'
pBI121-*CsaV3_5G039430M*	5'-ataattctgatttttctgGTCCCTCTTTACGAGACCGTCTT -3'	5'-cagaaaaatcagaattatGTAAGGGATGGCTTGGAGGG-3'
pFGC5941-*CsNPF4.4*	5'-tacaaatctatctctctcgagATGCAAAGAGATCATGAAGATATTTGT-3'	5'-gcccttgctcaccatggatccAGGCACTACAAGCTTAGAGTTTGAAG-3'
pAbAi-*MIR9748*	5'-cttgaattcgagctcggtaccCGTAAGAACCTGCCCTTGGG-3'	5'-atacagagcacatgcctcgagGAGTTAGCCGATGCTTATTCCC-3'
pGADT7-*CsbZIP2*	5'-gccatggaggccagtgaattcATGGGGATTCAAACTATGGGG-3'	5'-cagctcgagctcgatggatccTTAGAACGGCGCTGACGATG-3'
pGADT7-*CsMYB44*	5'-gccatggaggccagtgaattcATGGCGCTTACCCGTAAAGA-3'	5'-cagctcgagctcgatggatccTTAAAAGCTAACGTTCTTAATCCCAC-3'
pGADT7-*CsMYCI*	5'-gccatggaggccagtgaattcATGGGAAGGTCACCTTGTTGTG-3'	5'-cagctcgagctcgatggatccTTAGAAAATTGAAGAATCAGATGGAGA-3'
pGADT7-*CsHBP-1b*	5'-gccatggaggccagtgaattcATGGGTAGTAGAACAGTGAAGATTGG-3'	5'-cagctcgagctcgatggatccTCAATCTCGTGGCCGTGC-3'
pGADT7-*CsTGA2*	5'-gccatggaggccagtgaattcATGGCTACTGAGGGCAGTCCC-3'	5'-cagctcgagctcgatggatccTTACTCTCTGGGACGAGCAAGC-3'
pGADT7-*CsTGA2.2*	5'-gccatggaggccagtgaattcATGGTTCTGTATAAACTCTCAGTTATTATAGAG-3'	5'-cagctcgagctcgatggatccTCACTCTTTTGGGCGAGCAA-3'
pGADT7-*CsTGA10*	5'-gccatggaggccagtgaattcATGGCTTCTTCCAATATTAAAAACAG-3'	5'-cagctcgagctcgatggatccTTAATCTTGCCTTGGACGAGCC-3'
pGreenII0800-*MIR9748*	5'-ctatagggcgaattgggtaccTTCCAAGTTGTTCAAGAATAGTGGC-3'	5'-atcgataccgtcgacctcgagCATTCCGGATAACGCTTGCA-3'
pFGC5941-*CsbZIP2*	5'-tacaaatctatctctctcgagATGGGGATTCAAACTATGGGG-3'	5'-ggatccccgggtaccgagctcTTAGAACGGCGCTGACGATG-3'

Supplementary Table 2. Primers used for qPCR assays.

Gene name	Forward primer	Reverse primer
U6	5'-GGGGACATCCGATAAAATT-3'	5'-TGTGCGTGTCATCCTTGC-3'
miR9748	5'-GAGGAAGGTGAGGATGAC-3'	
CsaV3_5G039430	5'-TCACTCGTCGCCTCACCT-3'	5'-ATCCCTTCCACAGATTGCTT-3'
CsaV3_6G007840	5'-AAGCGATTCAGAAGCAGTAG-3'	5'-CAACGCCATTTCCAAGAT-3'
CsaV3_1G045520	5'-AGCGTTTATCAAAGTCAG-3'	5'-TCTTCATAAGTAGGCACAT-3'
CsaV3_7G029600	5'-AGCAGGGATGAAAGAGCA-3'	5'-TGGATTTAGCCCGAAGAG-3'
CsActin	5'-CAGGAATCCACGAAACTACT-3'	5'-AGACCCTCCAATCCAAACAC-3'
AtAOS	5'-AATCCCGCAGATACAAAG-3'	5'-TTCTCGTGAGATACCCAAT-3'
AtJAR1	5'-ATTACGCTATCTTCTGGG-3'	5'-GCATTACTTGGCTTCACA-3'
AtAOC4	5'-GGGCTGTTGTTCATTGTG-3'	5'-ATATCGGAAGGTGGAGTT-3'
AtLOX2	5'-TTCAGCCGTCTATGGTAA-3'	5'-TGTTCTGCGGTCTTATCT-3'
AtActin	5'-GGGGACATCCGATAAAATT-3'	5'-TGTGCGTGTCATCCTTGC-3'

Supplementary Table 3. Overview of raw and clean reads in WT and OE3 plants exposed to high temperature.

Sample Name	Total Raw Reads (M)	Total Clean Reads (M)	Total Clean Bases (Gb)	Clean Reads Q20 (%)	Clean Reads Q30 (%)	Clean Reads Ratio (%)
OE3-CK1	49.08	44.34	6.65	95.91	90.5	90.34
OE3-CK2	49.08	44.58	6.69	95.89	90.46	90.84
OE3-CK3	49.08	44.88	6.73	95.86	90.39	91.44
OE3-HT1	49.08	45.02	6.75	95.96	90.56	91.72
OE3-HT2	47.33	43.86	6.58	95.95	90.52	92.68
OE3-HT3	47.33	43.9	6.58	95.83	90.25	92.75
WT-CK1	49.08	44.32	6.65	95.98	90.66	90.3
WT-CK2	49.08	44.76	6.71	95.81	90.26	91.2
WT-CK3	49.08	44.63	6.69	95.69	90.03	90.93
WT-HT1	49.08	45.04	6.76	96	90.66	91.76
WT-HT2	49.08	44.7	6.71	95.94	90.53	91.08
WT-HT3	49.08	44.23	6.63	96.05	90.77	90.12

Supplementary Table 4. Mapping results of clean reads against the Arabidopsis genomic sequence.

Sample Name	Total Raw Reads (M)	Genome		Gene	
		Total Mapping (%)	Uniquely Mapping (%)	Total Mapping (%)	Uniquely Mapping (%)
OE3-CK1	44.34	94.74	92.63	92.38	82.72
OE3-CK2	44.58	94.76	92.71	91.95	82.65
OE3-CK3	44.88	94.79	92.66	91.87	82.09
OE3-HT1	45.02	94.83	92.00	87.88	83.51
OE3-HT2	43.86	95.35	92.85	89.17	84.99
OE3-HT3	43.9	95.13	92.50	88.51	84.45
WT-CK1	44.32	94.57	92.49	92.09	82.72
WT-CK2	44.76	94.33	92.21	91.75	81.73
WT-CK3	44.63	94.16	92.05	92.38	81.63
WT-HT1	45.04	95.23	92.85	89.29	84.2
WT-HT2	44.7	95.03	92.68	89.27	84.21
WT-HT3	44.23	94.78	92.36	88.98	83.93

Supplementary Table 5. Description of the cucumber *NPF4.4*.

Gene name	Accession number	Location in chromosome	CDS (bp)	Amino acid	MW (KDa)	pI
AtNPF4.4	AT1G33440	Chr1	1806	601	66.71	8.71
CsNPF4.4	CsaV3_5G039430	Chr5	1734	577	64.31	8.36

bp, base pair; MW, molecular weight; pI, isoelectric point.
